# Adjuvant Therapy for Attention in Children with ADHD Using Game-Type Digital Therapy

**DOI:** 10.3390/ijerph192214982

**Published:** 2022-11-14

**Authors:** Seon-Chil Kim, Hojun Lee, Hyun-Suk Lee, Gaeun Kim, Jeong-Heon Song

**Affiliations:** 1 Department of Biomedical Engineering, School of Medicine, Keimyung University, 1095 Dalgubeol-daero, Daegu 42601, Republic of Korea; 2 Department of Psychiatry, Keimyung University Dongsan Medical Center, Daegu 42601, Republic of Korea; 3 Department of Psychiatry, School of Medicine, Keimyung University, Daegu 42601, Republic of Korea; 4 Woori Children’s Development Clinic, Affiliated with Woori Soft, 175, Wolbae-ro, Dalseo-gu, Daegu 42781, Republic of Korea; 5 College of Nursing, Keimyung University, Daegu 42601, Republic of Korea; 6 Woorisoft Inc., 175, Wolbae-ro, Dalseo-gu, Daegu 42781, Republic of Korea

**Keywords:** attention deficit hyperactivity disorder (ADHD), game-based digital therapy (DTx), attention

## Abstract

Children diagnosed with attention deficit hyperactivity disorder (ADHD) require early intervention and sustained treatment. This study used a game-based digital treatment planning NeuroWorld DTx to analyze the impact on attention and cognitive function in children with ADHD. Thirty children diagnosed with ADHD were recruited and subjected to a four-week NeuroWorld DTx digital treatment. To assess the impact of the digital therapeutic therapy on children’s attention, we used the comprehension attention test (CAT) and Korean ADHD Rating Scale (K-ARS). Clinical global impression (CGI) and the Korean-child behavior checklist (K-CBCL 6–18) were used to examine the degree of improvement in ADHD. After four weeks, significant differences in the sensitivity and response style indices were noted, as compared with the baseline in the CAT test; in the case of K-ARS and CGI, a moderate decrease in ADHD was confirmed. The study achieved better results for the “total behavior problems” belonging to the K-CBCL assessment. Game-based digital therapy intervention can be a treatment method that elicits interest and satisfaction in children with ADHD and can be used as an adjunct to drug therapy to improve the quality of life and strengthen attention in children with symptoms of ADHD.

## 1. Introduction

The COVID-19 pandemic has, among its many direct and indirect effects on society, led to a mental health crisis among students [1]. Studies have reported that children and adolescents with existing psychiatric disorders are more adversely affected with mental health issues [2]. Psychiatric disorders can be categorized as internalizing and externalizing disorders [3]. Internalizing disorders include depressive disorders and anxiety disorders, whereas externalizing disorders include attention deficit hyperactivity disorder (ADHD), oppositional defiant disorder (ODD), and conduct disorder (CD) [4]. Among these, ADHD is a representative childhood disease, with inattention, hyperactivity, and impulsivity as the main symptoms [5,6]. The prevalence of ADHD was found to be 5–6% in the school-age group, and 3–7% of the American children were diagnosed with ADHD [7]. A significant number of children with ADHD are generally aggressive and have difficulty regulating their emotions in social and educational activities [8,9]. In addition, children with ADHD have many comorbidities, including ODD, depression, adjustment disorder, anxiety disorder, obsessive compulsive disorder, alcohol and substance abuse, and poor learning [10]. Early detection and treatment are of paramount importance given the seriousness of these complications.

The symptomatic treatment of ADHD is currently based on drug treatment, which significantly improves the core symptoms [11,12]. However, it is difficult to sufficiently control the core and additional symptoms of ADHD with medication alone [13]. In addition, owing to the adherence and rejection of ADHD children and their guardians to drugs, side effects, anxiety about drug treatment, interest in other non-pharmaceutical treatments that can supplement drug treatment is increasing [14]. Thus far, the representative non-drug treatment employed for ADHD is behavioral therapy; various methods such as play therapy, parent education, social skills training, and learning therapy are also being studied [15,16]. Recently, with the development of mobile-based software technology, digital therapy (DTx) adjuvant therapy has been developed, which is based on the use of digital devices. Digital therapy (DTx) is defined by the Digital Therapeutics Alliance as “evidence-based therapy driven by software to prevent, manage, or treat a medical disorder or disease” [17]. Through DTx, it is possible to improve adherence to prescribed drugs, monitor patients without time and place restrictions, and enable daily communication with patients, which can be significantly beneficial for chronic psychiatric disorders such as ADHD [18]. In addition, according to a recent trial investigating the effect of video-based gaming training on cognitive function and other neurological disease-related symptoms in patients with multiple sclerosis, video-based gaming training was effective for health-related areas such as gait, cognitive function, fatigue, and depression and was found to have almost the same effect as conventional treatment in terms of improving the quality of treatment [19]. Adjuvant therapy using these digital media can support patients who do not want standard treatment or those who want results that are not associated with many pharmacological effects [20]. In addition, in the case of mobile-based functional games for children of low ages, the high accessibility can help increase the interest in ADHD treatment learning and active motivation, leading to improvements in the therapeutic learning ability. Therefore, this study aimed to evaluate the effectiveness of a game-type NeuroWorld cognitive training program for children with ADHD using digital therapy.

## 2. Materials and Methods

### 2.1. Participants

Thirty children aged six to thirteen years participated in this study. The number of study participants was calculated using the software G*power (version 3.1.2) [21,22,23], with two-sided significance level (α) = 0.05, effect size (d) = 0.25, and power (1 − β) = 0.90. Although 26 participants were required, 30 were recruited, assuming a dropout rate of 20%. However, there were no dropouts in the actual study. The criteria for selecting the study participants included (1) those diagnosed with ADHD by one psychiatrist according to the DSM-5 diagnostic criteria of the Mental Health Diagnosis and Statistical Manual through the K-SADS-PL tool, (2) those with legal guardians who provided voluntary written consent, and (3) those who could follow the evaluation and guidelines of the research director. The exclusion criteria were as follows: (1) those with symptoms other than ADHD symptoms or accompanying symptoms; (2) those with conditions that affect the use of research products (physical deformities of the hand or wave, prosthetic limb); (3) those with significant symptoms including seizure disorders and reduced athletic ability; (4) those with color blindness; (5) those whose family members were already enrolled in the same study; and (6) those who were unsuitable for participating in the study. The study included 30 children; among these, 15 children received NeuroWorld digital therapy and conventional drug treatment through random assignment, and 15 children received conventional drug treatment alone. The age (t = 0.61; *p* > 0.05) and sex (t = 0.66; *p* > 0.05) of the two groups were not significantly different. Detailed information regarding the study participants is shown in Table 1. 

### 2.2. Study Design

**Figure 1:**
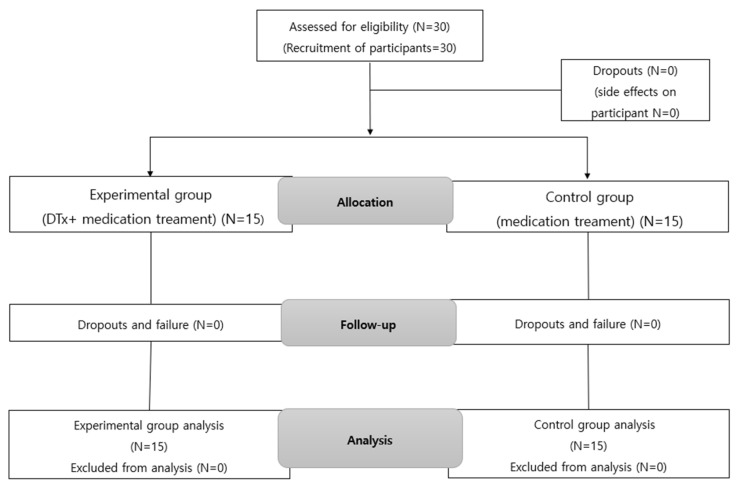
Flow diagram of study participants.

The experimental group received NeuroWorld DTx and ADHD medication treatments, whereas the control group received the ADHD medication treatment alone. This helped verify the effect of NeuroWorld DTx. The two groups were designed before and after the experiment, as shown in Table 2. The study period and contents are shown in Table 3, and the flow diagram of participants is presented in Figure 1.

### 2.3. Procedures 

This study was conducted from 19 January 2022 to 15 April 2022 at the Department of Mental Health Medicine, Keimyung University, Dongsan Hospital. In consideration of the ethical content of the subject, approval from the Institutional Bioethics Review Committee was obtained prior to data collection and evaluation. The study was performed after receiving approval (IRB No. 2021-10-080). The research participation agreement and description included information regarding the research purpose, participation procedure, participation risks and benefits, confidentiality, and not using the collected data for purposes other than research. After obtaining informed consent from both the participants and their guardians, the data collection and experiments were initiated. These data were kept obligatory, in accordance with the Bioethics Act.

### 2.4. Measures

#### 2.4.1. Comprehension Attention Test (CAT)

Attention was measured using the comprehension attention test (CAT), developed to evaluate various comprehensive types of attention and task execution functions for children and adolescents with ADHD [24,25]. The CAT consists of six tests: the visual selective attention task, auditory selective attention task, flanker task, sustained attention to response task, divided attention task, and spatial working memory task. Sustained attention was evaluated as restrained attention. In this study, for the evaluation of selective attention, four CAT test elements were selected and evaluated as follows: visual simple selection attention, auditory simple selection attention, interference selection attention, and inhibitory persistence attention. To examine the subject’s response to learning tools through the four CAT test elements, the sensitivity coefficient and rate of change of the sensitivity coefficient were calculated based on the ability to discriminate between target and non-target stimuli [26]. In general, when the sensitivity coefficient is two or higher, the target and non-target stimuli are effectively distinguished [27]. In addition, the response style of attention was calculated. Changes in the response style index between the two groups were compared by setting the values of each subject to one, to determine whether improvements were achieved compared with the baseline after 4 weeks. The values were set to −1 when worsening and 0 when there was no change. The reliability was Cronbach α = 0.79 [28].

#### 2.4.2. Korean Version of Attention Deficit Hyperactivity Disorder (Korean ADHD Rating Scale, K-ARS)

The degree of ADHD was measured using the attention deficit hyperactivity disorder rating scale, based on the DSM-IV ADHD diagnostic criteria [29,30]. The evaluation tool consists of 18 items in two areas: attention deficit (inattention) and hyper impulsivity. Each item is rated on a 4-point Likert scale, with 0 points for “not at all”, 1 point for “sometimes”, 2 points for “often”, and 3 points for “very often”, depending on the frequency of the child’s problem behavior. The evaluated total score was 0–54 points. The higher the score, the more severe the attention deficit hyperactivity disorder [31].

#### 2.4.3. Clinical Global Impression (CGI)

The overall clinical impression scale was used to evaluate the outcome of the worsening or improvement of symptoms before and after treatment [32,33]. This tool evaluates clinical severity (clinical global impression-severity, CGI-S) and improvement (clinical global impression-improvement, CGI-I) at the time of evaluation by a clinician. On a 7-point scale, 1 point means normal (no mental illness), 2 points means borderline mental illness, 3 points means mildly ill, 4 points means moderately ill, 5 points markedly ill, 6 points means severely ill, and 7 points means extremely ill. The higher the score, the worse the situation, and the lower the score, the better the situation [34,35]. The CGI score used in this study was evaluated and observed by the psychiatrist directly. The rate of change in the CGI score was calculated as ((Baseline CGI) − (Follow #2 CGI))/(Baseline CGI) 100.

#### 2.4.4. Korean Child Behavior Checklist (K-CBCL 6–18)

A tool standardized by K-CBCL 6–18, developed to evaluate the level of problem behaviors and social adaptation of children, was used for the parents of children and adolescents aged 6–18 years [36,37]. This tool is divided into two scales: problem behavior and social adaptability. Problem behaviors include internalizing, externalizing, and total problem behaviors. Each item is evaluated on a 3-point scale ranging from 0 (not applicable at all) to 2 (often), where a higher score indicates higher problem behavior tendencies. The reliability was Cronbach’s α = 0.62–0.86 [38].

### 2.5. Experimental Tool

**Figure 2:**
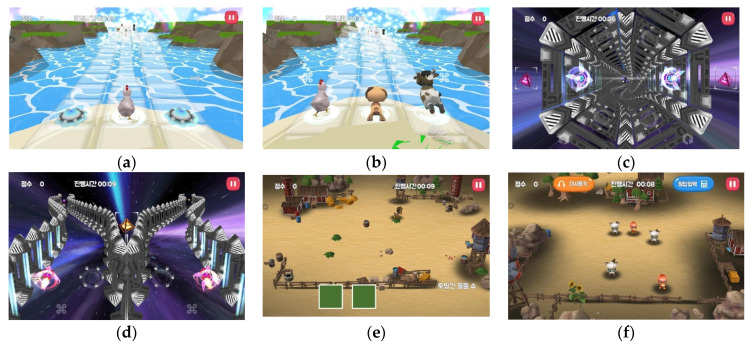
NeuroWorld DTx configuration and game screens. (**a**) Attention 1: Breathtaking glass bridge. (**b**) Attention 2: Find a friend in glass bridge. (**c**) Attention 3: Space travel in vortex. (**d**) Attention 4: Space travel. (**e**) Working memory 1: Animals out of the yard. (**f**) Working memory 2: Escape from the yard.

In this study, a tablet PC installed with NeuroWorld DTx, an AI-based attention and working memory improvement training program, was provided to each child participant. NeuroWorld DTx is a game-based program designed to enable home participation and therapy for attention deficit and impulsivity. NeuroWorld DTx is a game-based cognitive therapy software developed by Author and head of research center in Woorisoft Co., Ltd., (Daegu, Korea) of J.H.S. It is designed to enable home participation and therapy as an adjunct treatment for ADHD such as attention deficit and impulsivity. This study uses an unverified digital therapeutic agent is currently in the R&D stage. Therefore, considering that the participants in the study were vulnerable children between the ages of 6 and 13, the necessity and procedures of the program were explained to the guardians of the children and their prior consent was obtained. It can be used as an adjunct treatment for ADHD. NeuroWorld DTx explores the possibility of digital therapy as a mobile-based functional game for children with ADHD and evaluates whether digital therapy can be expected to improve attention and reduce ADHD symptoms. Notably, NeuroWorld DTx controls the excessive use of digital media to prevent side effects by automatically restricting the usage time to 30 min a day. NeuroWorld DTx comprises six items, four sets of attentiveness and two sets of working memory, as shown in Table 4 and Figure 2.

### 2.6. Statistical Analysis

The statistical processing of data in this study was analyzed at a significance level of *p* < 0.05 using SPSS for Windows 23.0 (SPSS Inc., Chicago, IL, USA). The mean and standard deviation of the basic data were calculated and analyzed using a paired *t*-test to verify the homogeneity of the baselines for the two groups. In addition, the test results obtained in week 4 for the experimental and control groups were then compared with the baseline. The analysis of covariance (ANCOVA) was performed on the CAT and CGI scales. Korean ADHD Rating Scale (K-ARS) and Korean-Child Behavior Checklist (K-CBCL) were measured using the repeated measures ANOVA. The homogeneity test for the general characteristics of the experimental and control groups was conducted using Pearson and Fisher’s exact tests. Further, the *t*-test was employed as the homogeneity test for the dependent variable, when the normal distribution was satisfied, whereas the Mann–Whitney U test was used when the normal distribution was not satisfied [39,40].

The NeuroWorld content satisfaction survey, which was used by both participants and parents as a training program, was conducted through a direct questionnaire. The percentages were calculated and analyzed. The efficacy evaluation was analyzed using modified intention-to-treat (ITT) and per-protocol (PP) analysis; however, in principle, the final decision of the study results was based on the modified ITT analysis results.

The modified ITT analysis group was assigned to the NeuroWorld DTx group after randomization, and data collection on the primary efficacy endpoint was possible after baseline. Among the modified ITT analysis participants, those without significant violations (dropout, violation of selection/exclusion criteria, violation of randomization, etc.) were used for the PP analysis under the study protocol procedure. The last observation carried forward (LOCF) method was used; this approach analyzes the data measured at the last visit by replacing the data measured at the last visit with the corresponding time point if dropouts occurred at a certain point in time for the efficacy endpoint. The safety evaluation involved checking whether all the data were coded and stored on an external hard drive separate from the computer for all the participants enrolled in this study and those who received NeuroWorld DTx at least once. To protect the confidentiality of the data, the personally identifiable information of the collected data was coded, the collected data were not used for purposes other than research, and the survey data were crushed and discarded after the data retention period, according to the Bioethics Act (3 years for consent; 5 years for other data).

## 3. Results

### 3.1. Neuro-World DTx Program Attention Improvement

#### 3.1.1. Comprehension Attention Test (CAT)

**Figure 3:**
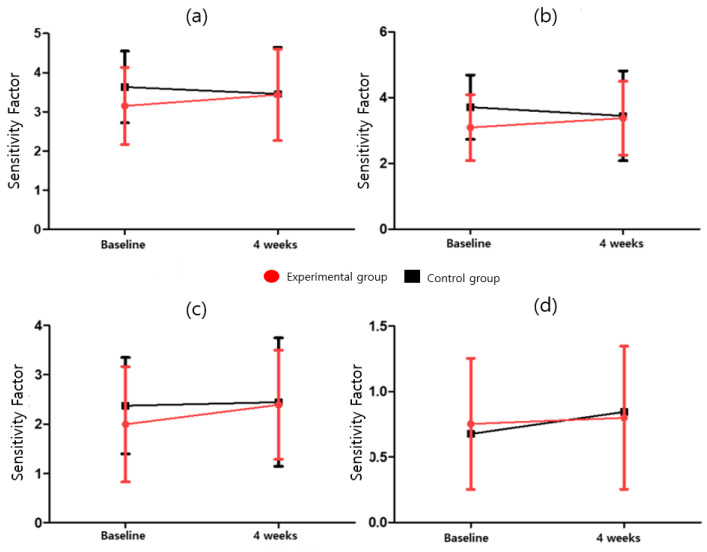
Attention task sensitivity factor for (**a**) visual selective attention task, (**b**) auditory selective attention task, (**c**) sustained attention to response task, and (**d**) Flanker task.

**Figure 4:**
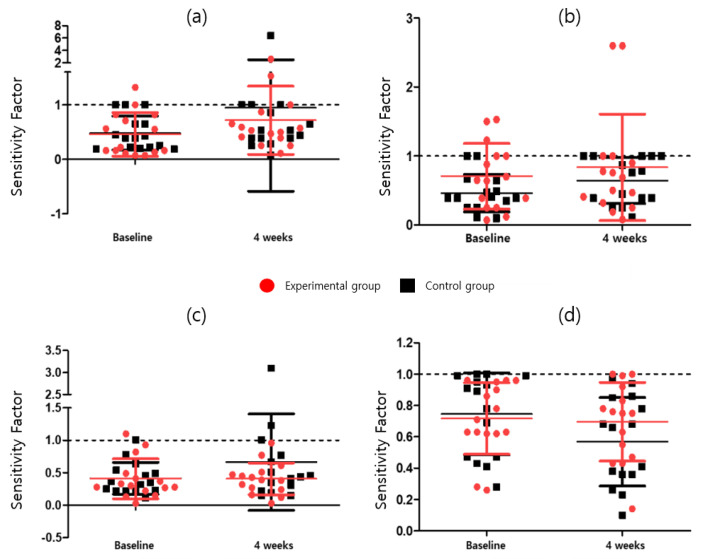
Attention task response style index for (**a**) visual selective attention task, (**b**) auditory selective attention task, (**c**) sustained attention to response task, and (**d**) Flanker task.

In the CAT, the attention task sensitivity factor and attention task response style index were analyzed. The attention task sensitivity factor includes the visual selective attention task (F = 2.042; *p* = 0.164), auditory selective attention task (F = 2.856; *p* = 0.103); Flanker task (F = 0.285; *p* = 0.598), and sustained attention to response task (F = 0.530; *p* = 0.473). The attention task response style index includes the visual selective attention task (F = 0.245; *p* = 0.624), auditory selective attention task (F = 0.374; *p* = 0.546), Flanker task (F = 6.142; *p* = 0.020), and the sustained attention to response task (F = 1.954; *p* = 0.174). After four weeks of program intervention, Attention Task_Sensitivity Factor was 2 or higher in both the experimental and control groups in Visual Selective Attention Task, Auditory Selective Attention Task, and Sustained Attention to Response Task except for Flanker Task, and for Auditory Selective Attention Task, four weeks compared to baseline in the experimental group Afterwards, there was a significant increase (3.09 ± 1.00 vs. 3.37 ± 1.12, *p* = 0.037), although no significant difference between the experimental and control groups was observed. Among the Attention Task_Sensitivity Factors, only the experimental group was significant in the Auditory Selective Attention Task (t = −2.299, *p* < 0.05). There was no statistically significant difference between the groups in the ANCOVA analysis with baseline correction in the Attention Task_Sensitivity Factor change rate. The Attention task_Response Style Index showed a significant difference (*p* = 0.020) between the experimental (0.70 ± 0.25) and control (0.57 ± 0.28) groups owing to the baseline-corrected ANCOVA in the Flanker task. In the Response Style Index change rate, the number of cases in which the Flanker Task index worsened was 14 (93.3%) in the control group compared to 8 (53.3%) in the experimental group. There was a statistically significant difference (*p* = 0.020) between the experimental group (−1.70 ± 29.14) and the control group (−25.43 ± 23.42) in the Flanker Task’s Response Style Index change rate. Detailed results are presented in Table 5 and Table 9, Figure 3 and Figure 4.

#### 3.1.2. Evaluation Results for Attention Deficit Hyperactivity Disorder (Korean ADHD Rating Scale, K-ARS)

**Figure 5:**
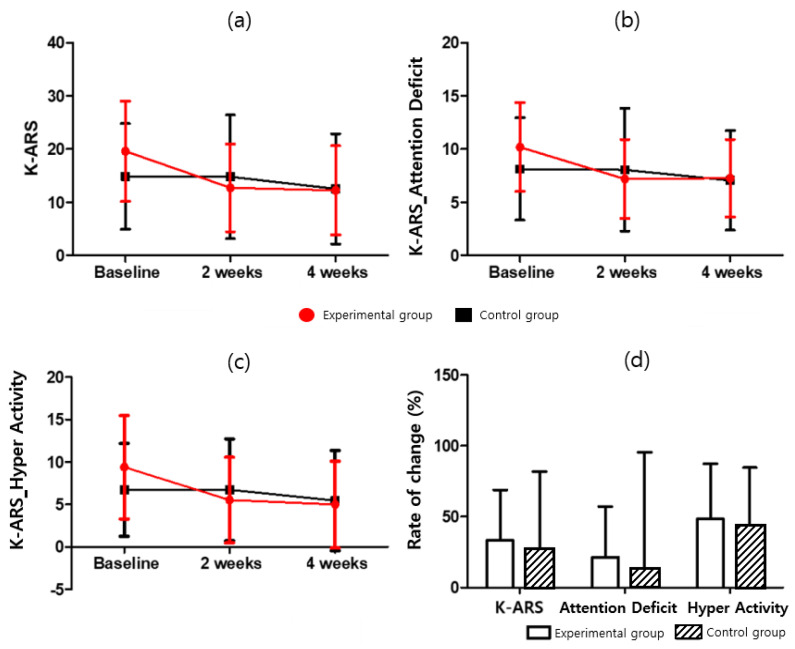
K-ARS score for (**a**) K-ARS, (**b**) K-ARS-Attention Deficit, (**c**) K-ARS-Hyper Activity, and (**d**) K-ARS-Rate of change.

For the ADHD rating scale, the total score, inattention, and hyperimpulsivity scores were evaluated as the rate of change, as compared with the baseline, at two weeks and four weeks after the experiment. The results for K-ARS (F = 0.069; *p* = 0.795), K-ARS-Attention Deficit (F = 0.118; *p* = 0.773), and K-ARS-Hyper Activity (F = 0.002; *p* = 0.963) are presented herein. After four weeks of intervention, the total K-ARS score, Attention Deficit, and Hyper Activity of the experimental group were 12.27 ± 8.39, 7.27 ± 3.63, and 5.00 ± 5.11, respectively, which showed a statistically significant decrease compared to baseline (t = 3.281, t = 2.683, t= 3.491, *p* < *0*.05). However, they were not significant in the control group (t = 0.842, t = 0.776, t = 0.857, respectively, *p* > *0*.05). For additional details, please refer to Table 6 and Table 9, and Figure 5.

#### 3.1.3. Clinical Global Impression (CGI)

**Figure 6:**
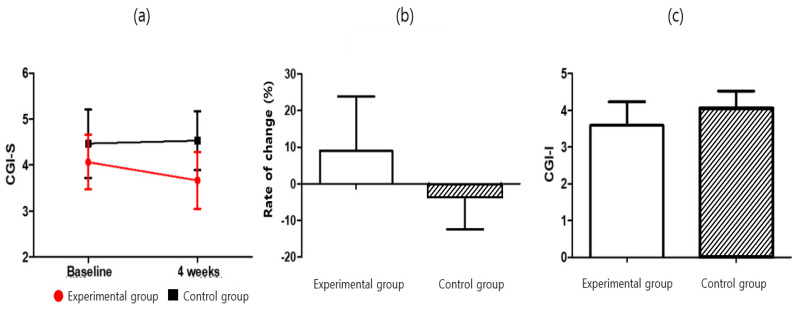
CGI score for (**a**) CGI-S, (**b**) CGI-S-Rate of change, and (**c**) CGI-I.

The clinical global impression (CGI) was used to evaluate the degree of worsening or improvement of the symptoms before and after treatment. The severity of symptoms (CGI-S) was significantly lower in the experimental group (3.67 ± 0.62) than in the control group (4.53 ± 0.64) after four weeks of intervention (*p* = 0.001). Furthermore, significant difference between the two groups in the rate of change (*p*= 0.023) was observed. For the experimental group, the measured severity of the symptoms (CGI-Severity, which was F = 11.164 and *p* = 0.002) was significantly lower after four weeks of intervention (t = 2.449; *p* < 0.05). The control CGI-S score increased considerably but was not significant (t = −0.564; *p* > 0.05). The difference between the two groups was also significant (F = 11.164; *p* < 0.05). The degree of symptom improvement (CGI-I) was significantly lower in the experimental group (3.60 ± 0.63) than in the control group (4.07 ± 0.46) (*p* = 0.029). For additional details, please refer to Table 7 and Table 9, and Figure 6.

#### 3.1.4. Korean-Child Behavior Checklist (K-CBCL 6–18)

**Figure 7:**
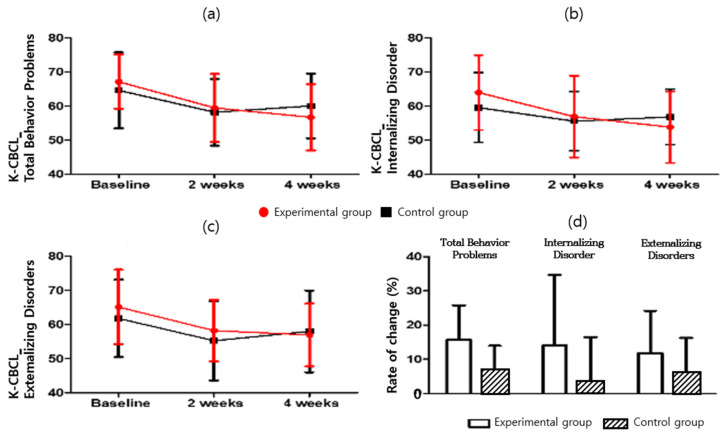
K-CBCL score for (**a**) K-CBCL_Total Behavior Problems, (**b**) K-CBCL_Internalizing Disorder, (**c**) K-CBCL_Externalizing Disorders, and (**d**) K-CBCL_Rate of change.

For the K-CBCL problem behavior and social adaptation, the scores and change rates of total problem behavior, internalization, and externalization in K-CBCL were compared (Table 8 and Table 9 and Figure 7). The results for K-CBCL_Total Behavior Problems (F = 0.002; *p* = 0.963), K-CBCL_Internalizing Disorder (F = 0.076; *p* = 0.785), and K-CBCL_Externalizing Disorders (F = 0.225; *p* = 0.639) are presented herein. Statistically significant difference between the experimental group (15.66 ± 10.12) and the control group (6.68 ± 7.20) was observed in the Total Behavior Problems score change rate (*p* = 0.009). The experimental group showed a significant decrease (t = 6.064; *p* < 0.05) in the K-CBCL total problem behavior score. By contrast, the control group’s K-CBCL total problem behavior score increased significantly (t = 3.740; *p* < 0.05). There was no significant difference between the two groups (F = 0.002; *p* > 0.05). As a result of measuring the internalization score in the internalization and externalization rate of change, the experimental group showed a significant decrease (t = 3.527; *p* < 0.05). The control group had an increased score (t = 1.377; *p* > 0.05), and there was no difference between the two groups (F = 0.076; *p* > 0.05). In addition, although the externalization score decreased significantly for the experimental group (t = 3.615; *p* < 0.05), the control group showed a significantly increased score (t = 2.492; *p* < 0.05). There was no difference between the two groups (F = 0.225; *p* > 0.05).

## 4. Satisfaction Survey Results

After applying NeuroWorld DTx, a satisfaction survey was conducted among the children and parents (Table 10). The children’s overall satisfaction results showed that 60% of them were satisfied, whereas 40% found it normal. The parents’ overall satisfaction results showed that 73% of them were satisfied, whereas 26% found it normal. When asked if they would re-participate in the NeuroWorld DTx intervention, 80% said yes, 6.6% said no, and 13.3% provided other answers.

## 5. Discussion

This study investigated the effects of digital therapy on the attention function and symptoms of children diagnosed with ADHD. Clinical efficacy and safety were evaluated prospectively, on a single-center and standard-of-care basis. When combined with drug therapy, NeuroWorld DTx treatment led to an improved treatment effect in children with ADHD, as compared with the baseline. In this study, game-based digitization using NeuroWorld DTx was used. In the treatment regimen, the results of the four tests could summarize the attention of the study participants. CAT was analyzed using two indicators: sensitivity coefficient and response style. There was no significant difference in the scores between the experimental and control groups. However, four weeks after auditory simple selection attention, the sensitivity value of the experimental group showed a significant increase compared with the baseline, and the interference selection attention showed improvements in terms of the response style index. In the case of the attention deficit hyperactivity disorder (K-ARS), there was no significant difference in the K-ARS score and rate of change at two weeks, as compared with the baseline. However, after four weeks, a significant decrease in the degree of attention deficit hyperactivity disorder was confirmed in the experimental group, relative to the baseline. Significant effects in terms of the degree of severity and improvement (CGI) were also confirmed when NeuroWorld DTx and drug treatment were combined. In the case of the K-CBCL results, there were significant differences in the total scores for problem behavior, internalization, and externalization in the experimental group after four weeks of contrast intervention, as compared with the baseline. These results confirm that NeuroWorld DTx relieves the problem behavior syndrome. The improvement in ADHD symptoms after NeuroWorld DTx treatment achieved in this study was related to the existing digital therapy intervention, which is also valid for a previous case [41].

Previous digital therapy intervention studies have shown that digital intervention can be applied to standard care without additional safety concerns and can help reduce the barriers to access, which are inherent in other forms of behavioral or non-pharmacological interventions [42]. In particular, game-type digital therapeutics such as AKL-TO1 have been proven to improve measured inattention in ADHD patients, while presenting minimal side effects [43]. It is also very effective as a home treatment tool or learning material in the case of exceptional social circumstances, such as the isolation due to the COVID-19 pandemic. Existing studies indicate that providing digital treatment interventions through mobile devices can increase the access to treatment and reduce treatment costs, which, in turn, can help improve compliance [44]. NeuroWorld DTx research offers the advantage of improving the objectively measured inattentiveness of pediatric ADHD patients and increasing compliance through greater motivation and the active participation of children, including those refusing to participate in the study, or not giving up or missing out on participants. In the future, the transformation and expansion of game-type content, such as group therapy, can be configured to induce various social relationships [45,46].

Children with ADHD symptoms are usually preschoolers or young children. They exhibit a lack of motivation to solve problems, and their language is not fully developed. Thus, they are highly dependent on the environment and objects [47,48]. According to updated clinical practice guidelines from the American academy of pediatrics (AAP), symptomatic treatment to children with ADHD (6–11 years of age) entails medication and behavioral therapy. However, owing to the current shortage of mental health professionals, medication is more prevalent [49]. Therefore, to overcome these limitations, game-based treatment contents such as NeuroWorld DTx can help administer continuous concentration and memory training. It is important to elicit interest and motivation and also enhance attention among children with ADHD symptoms. NeuroWorld DTx is also expected to play a role in reducing drug dependency [50,51]. According to previous studies, in the case of ADHD, there is a higher risk of comorbidity in girls, as compared with boys, diagnosed with ADHD depending on the environment [52]. Owing to the nature of psychiatric research on children and adolescents, it is difficult to obtain a sample of study participants, and it is known that the prevalence of ADHD in boys is more than twice that in girls; thus, the number of actual hospital visits is considerably lower for girls than for boys. Therefore, there were more male participants in the current study. However, there was no significant difference in the gender distribution between the experimental and control groups participating in the experiments. Therefore, this study shows that digital therapy can achieve the same effect as drugs, psychological counseling, education, behavioral therapy, face-to-face educational intervention, family therapy, and sociality training, and that it can be adopted to realize therapeutic effects without gender differences [53,54].

To prevent the negative effects of continuously using smartphones and digital media, the learning method selected in this study stipulated a usage time. This control is effective for preventing the abuse of functional game contents. Active intervention is considered to control the safety of these tools [55].

A typical cognitive training program using digital media in the treatment process for children with ADHD symptoms is Endeavor Rx, which is a game software developed in the United States for the ADHD treatment of children aged 8–12 years [56]. Endeavor Rx is approved by the U.S. Food and Drug Administration (FDA) as a digital therapeutic. It has been demonstrated that game software can be used to improve cognitive function. In addition, it can be employed without space restrictions. Therefore, it can help compensate for the shortcomings of traditional cognitive training programs [57]. In a reality where drug treatment for children with ADHD is preceded, digital game-type content presented as an adjunct to treatment can increase the accessibility and effectiveness of drug treatments. With the development of current medical technology, treatment with drugs is the best for ADHD, but it is necessary to develop a treatment method that can replace drugs. Future research on Neuro World requires improvement in the size of the study to investigate the effectiveness of digital therapy for children who have never used drugs.

Nevertheless, this study has certain limitations. First, the participants included 30 children diagnosed with ADHD; here, the ADHD severity was considered as heterogeneous because the study was conducted on both patients who were previously diagnosed with ADHD and those who were diagnosed with ADHD for the first time. Second, only the short-term effects of digital therapy were analyzed. Although the analyses in this study afforded statistically significant results, observational studies over longer periods are required to show clinically significant results. Therefore, owing to the small number of participants and the short study period, there are limitations to various experimental factors. Hence, future studies considering the various long-term effects of digital therapy are required. Third, in this study, the age of children participating in the experiment was designated as between 6 and 13 years. Generally, 6 to 12 years of age is classified as childhood in the field of pediatric psychiatry, and those aged 13 to 18 are classified as adolescent. Therefore, in pediatric research, in general, the distribution of subjects for children’s studies is set from 6 to 12 years old, and the age range of participants in this study is also set from 6 to less than 13 years old, so the variables according to age change are not shown in detail. Therefore, in future, it is necessary to subdivide the age group and conduct a study to analyze the effectiveness according to age. Despite these limitations, NeuroWorld, which was used as an adjuvant therapy for drug treatment, showed a significant effect when combined with standard treatment. In addition, both the children and parents were satisfied with the NeuroWorld DTx digital therapy intervention and expressed their intention to re-participate. These findings suggest that the NeuroWorld Digital Therapy Program induces positive behavioral changes in children with ADHD. This also indicates its potential for use as an alternative treatment approach. In particular, it can be used as a therapeutic agent because of its excellent accessibility to children who miss the treatment period, despite the need for continuous treatment, owing to the spread of the COVID-19 pandemic. Therefore, in the future, digital adjuvant therapy and drug therapy can be used in parallel to improve the quality of life of children with ADHD.

## 6. Conclusions

This study investigated the effect of a digital adjuvant therapy, NeuroWorld DTx, on the attention and working memory of children diagnosed with ADHD, as well as possibility of combining this approach with conventional drug treatment. Based on the CAT change rate, it was confirmed that the participants of the experimental group showed improved sensitivity and response style indexes, as compared with the control group. The rate of change in the K-ARS total score, inattention, and hyperimpulsivity scores significantly decreased for the experimental group after four weeks. In addition, when NeuroWorld DTx and the standard treatment were combined, a significant decrease in the CGI-S severity evaluation was noted, thereby suggesting the effectiveness of NeuroWorld DTx. NeuroWorld DTx was found to be effective as a digital therapy adjuvant therapy along with drug treatment; thus, it shows potential for use as a digital adjuvant therapy. Current research indicates that drug therapy is the optimal treatment for children with ADHD. However, convenient and effective treatment requires the development of alternative methods such as NeuroWorld DTx. Future research should focus on controlled trials with a large number of children, which will help evaluate NeuroWorld DTx’s effects on a variety of children with ADHD symptoms.

## Figures and Tables

**Table 1:** Characteristics of study participants.

Group	Diagnosis	Mean Age (SD)	Gender (M/F)	NeuroWorld Experience	Accompanying Symptoms (Number of People)
Experimental group (*n* = 15)	ADHD	9.27 (1.62)	12/3	None	Tick-Symptom (2)
Control group (*n* = 15)		8.93 (1.91)	11/4	None	None

**Table 2:** Study design model.

Group	Pre-Inspection	Experimental Treatment	Post Inspection
Experimental group (*n* = 15)	Q1	X	Q2
Control group (*n* = 15)	Q1		Q2

Q1: Preliminary examination baseline (Physical examination, CAT, K-ARS, CGI, K-CBCL). Q2: Post examination after four weeks (Physical examination, CAT, K-ARS, CGI, K-CBCL). X: NeuroWorld DTx.

**Table 3:** Research period and contents.

Procedure	Study Period	Research Content
Recruitment of research subjects	1 January 2022– 10 March 2022	Mental health specialist treatment
Baseline	19 January 2022– 10 March 2022	Physical examination, CAT, K-ARS, CGI, K-CBCL (pre-inspection)
Interventional study	20 January 2022– 14 April 2022	NeuroWorld DTx applied 20 times
Maintain	21 February 2022– 15 April 2022	Physical examination, CAT, K-ARS, CGI, K-CBCL (Post-Inspection)

**Table 4:** Composition of the Neuro-World DTx program.

Composition		Content
Attention	Breathtaking glass bridge	A game of recognizing approaching animal types, determining their order and shape, and matching them
	Find a friend in glass bridge	The same game of matching animals of the same type, but it requires a bit more attention than “Breathtaking glass bridge”
	Space travel in vortex	A form of avoiding obstacles by grasping the path forward and the path of obstacles
	Space travel	Requires judgment and high attention in the form of manipulating the screen by focusing on two paths
Working memory	Animals out of the yard	A game that requires memory by listening to sounds and entering the number and type of animals
	Escape from the yard	A game of remembering the number of animals that appear on the screen and then disappeared

**Table 5:** Effect on attention function and symptoms (CAT).

			Experimental Group (*n* = 15)		Control Group (*n* = 15)		*p*-Value
			Mean or *n*	SD or %	Mean or *n*	SD or %	
Attention Task Sensitivity Factor	Visual selective attention task	Baseline	3.15	0.99	3.63	0.92	0.175 °
		4 weeks	3.43	1.17	3.46	1.19	0.958 °
		*p*-value	0.099 °°		0.467 °°		0.164 °°°
		Rate of change	10.69	34.83	−4.38	25.83	0.189 °
	Auditory selective attention task	Baseline	3.09	1.00	3.71	0.98	0.098 °
		4 weeks	3.37	1.12	3.44	1.37	0.879 °
		*p*-value	0.037 °°		0.335 °°		0.103 °°°
		Rate of change	9.18	15.78	−6.58	30.48	0.086 °
	Flanker task	Baseline	0.75	0.50	0.68	0.54	0.690 °
		4 weeks	0.80	0.55	0.84	0.89	0.869 °
		*p*-value	0.635 °°		0.380 °°		0.598 °°°
		Rate of change	12.94	76.89	67.45	400.94	0.609 °
	Sustained attention to response task	Baseline	2.00	1.17	2.37	0.98	0.348 °
		4 weeks	2.39	1.11	2.45	1.30	0.903 °
		*p*-value	0.069 °°		0.782 °°		0.473 °°°
		Rate of change	4.08	226.10	9.46	44.76	0.928 °
Attention Task Response Style Index	Visual selective attention task	Baseline	0.46	0.40	0.48	0.31	0.851 °
		4 weeks	0.71	0.63	0.95	1.53	0.594 °
		*p*-value	0.173 °°		0.220 °°		0.624 °°°
		Exacerbation	5	33.3	4	26.7	0.574 °°°°
		No Change	0	0.0	1	6.7	
		Improvement	10	66.7	10	66.7	
		Rate of change	208.34	353.01	83.52	150.40	0.218 °
	Auditory selective attention task	Baseline	0.71	0.47	0.46	0.27	0.093 °
		4 weeks	0.84	0.77	0.64	0.33	0.380 °
		*p*-value	0.560 °°		0.104 °°		0.546 °°°
		Exacerbation	7	46.7	4	26.7	0.361 °°°
		No Change	0	0.0	1	6.7	
		Improvement	8	53.3	10	66.7	
		Rate of change	74.04	169.66	120.91	244.91	0.547 °
	Flanker task	Baseline	0.72	0.23	0.75	0.26	0.746 °
		4 weeks	0.70	0.25	0.57	0.28	0.203 °
		*p*-value	0.630 °°		0.001 °°		0.020 °°°
		Exacerbation	8	53.3	14	93.3	0.035 °°°°
		No Change	0	0.0	0	0.0	
		Improvement	7	46.7	1	6.7	
		Rate of change	−1.70	29.14	−25.43	23.42	0.020 °
	Sustained attention to response task	Baseline	0.41	0.31	0.41	0.25	0.948 °
		4 weeks	0.41	0.24	0.66	0.74	0.213 °
		*p*-value	1 °°		0.147 °°		0.174 °°°
		Exacerbation	9	60.0	8	53.3	>0.05 °°°°
		No Change	0	0.0	0	0	
		Improvement	6	40.0	7	46.7	
		Rate of change	137.04	504.71	104.66	274.73	0.829 °

° Student *t*-test, °° Baseline vs. 4 weeks paired *t*-test, °°° ANCOVA, °°°° Chi-square test.

**Table 6:** Effect on attention function and symptoms (K-ARS).

			Experimental Group (*n* = 15)		Control Group (*n* = 15)		*p*-Value
			Mean or *n*	SD or %	Mean or *n*	SD or %	
ADHD Rating	K-ARS	Baseline	19.60	9.41	14.87	9.91	0.190 °
		2 weeks	12.73	8.25	14.80	11.61	0.579 °
		4 weeks	12.27	8.39	12.53	10.35	0.939 °
		*p*-value	0.005 °°		0.414 °°		0.795 °°°
		Rate of change	33.14	35.75	27.16	54.69	0.729 °
	K-ARS-Attention Deficit	Baseline	10.20	4.18	8.13	4.81	0.219 °
		2 weeks	7.20	3.71	8.07	5.76	0.628 °
		4 weeks	7.27	3.63	7.07	4.68	0.897 °
		*p*-value	0.018 °°		0.451 °°		0.733 °°°
		Rate of change	21.37	35.80	12.10	83.47	0.697 °
	K-ARS Hyper Activity	Baseline	9.40	6.09	6.73	5.47	0.218 °
		2 weeks	5.53	5.04	6.73	5.99	0.558 °
		4 weeks	5.00	5.11	5.47	5.89	0.818 °
		*p*-value	0.004 °°		0.406 °°		0.856 °°°
		Rate of change	48.38	38.93	43.45	41.30	0.743 °

° Student *t*-test, °° Baseline vs. 4 weeks paired *t*-test, °°° Repeated measure ANOVA.

**Table 7:** Effect on attention function and symptoms (CGI).

			Experiment Group (*n* = 15)		Control Group (*n* = 15)		*p*-Value
			Mean or *n*	SD or %	Mean or *n*	SD or %	
Clinical Severity and Improvement	CGI- Severity	Baseline	4.07	0.59	4.47	0.74	0.115 °
		4 weeks	3.67	0.62	4.53	0.64	0.001 °
		*p*-value	0.028 °°		0.582 °°		0.002 °°°
		Rate of change	9.00	14.90	−2.22	10.19	0.023 °
	CGI- Improvement	4 weeks	3.60	0.63	4.07	0.46	0.029 °

° Student *t*-test, °° Baseline vs. 4 weeks paired *t*-test, °°° ANCOVA.

**Table 8:** Effect on attention function and symptoms (K-CBCL).

			Experimental Group (*n* = 15)		Control Group (*n* = 15)		*p*-Value
			Mean or *n*	SD or %	Mean or *n*	SD or %	
Behavior Problems and Social Competence	K-CBCL _Total Behavior Problems	Baseline	67.13	7.95	64.60	11.10	0.478 °
		2 weeks	59.40	9.98	58.13	9.79	0.728 °
		4 weeks	56.67	9.70	60.00	9.49	0.349 °
		*p*-value	>0.05 °°		0.002 °°		0.963 °°°
		Rate of change	15.66	10.12	6.68	7.20	0.009 °
	K-CBCL _Internalizing Disorder	Baseline	63.93	11.00	59.53	10.20	0.266 °
		2 weeks	56.87	11.98	55.53	8.67	0.730 °
		4 weeks	53.80	10.52	56.80	8.13	0.389 °
		*p*-value	0.003 °°		0.190 °°		0.785 °°°
		Rate of change	13.99	20.66	3.35	13.10	0.103 °
	K-CBCL _Externalizing Disorders	Baseline	65.13	10.89	61.80	11.35	0.419 °
		2 weeks	58.20	9.03	55.27	11.63	0.447 °
		4 weeks	56.93	9.18	58.00	11.96	0.786 °
		*p*-value	0.003 °°		0.026 °°		0.639 °°°
		Rate of change	11.73	12.45	6.07	10.15	0.183 °

° Student *t*-test, °° Baseline vs. 4 weeks paired *t*-test, °°° Repeated measure ANOVA.

**Table 9:** Data Analysis Results.

	F-Value	*p*-Value		t-Value Experimental Group	*p*-Value Experimental Group	t-Value Control Group	*p*-Value Control Group
K-ARS	0.069	0.795	F-value: baseline, follow 1, 2 Repeated measure ANOVA	3.281	0.005	0.842	0.414
K-ARS Attention Deficit	0.118	0.733		2.683	0.018	0.776	0.451
K-ARS Hyper Activity	0.034	0.856		3.491	0.004	0.857	0.406
K-CBCL _Total Behavior Problems	0.002	0.963		6.064	0.000	3.740	0.002
K-CBCL_ Internalizing Disorder	0.076	0.785		3.527	0.003	1.377	0.190
K-CBCL Extemalizing Disorders	0.225	0.639		3.615	0.003	2.492	0.026
CAT _Visual Selective Attention Task_Sensitivity Factor	2.042	0.164	F-value: baseline, follow 2 baseline ANCOVA	−1.767	0.099	0.748	0.467
CAT_Visual Selective Attention Task_Response Style Index	0.245	0.624		−1.436	0.173	−1.283	0.220
CAT_Auditory Selective Attention Task__Sensitivity Factor	2.856	0.103		−2.299	0.037	0.998	0.335
CAT_Auditory Selective Attention Task_Response Style Index	0.374	0.546		−0.596	0.560	−1.739	0.104
CAT_Flanker Task_Sensitivity Factor	0.285	0.598		−1.966	0.069	−0.282	0.782
CAT_Flanker Task_Response Style Index	6.142	0.020		0.000	1.000	−1.536	0.147
CAT_Sustained Attention to Response Task_Sensitivity Factor	0.530	0.473		−0.485	0.635	−0.906	0.380
CAT_Sustained Attention to Response Task_Response Style Index	1.954	0.174		0.493	0.630	4.053	0.001
CGI-S	11.164	0.002		2.449	0.028	−0.564	0.582
				baseline v.s. follow 2 paired *t*-test			

**Table 10:** Responses of parents and children to research questionnaire.

	Answer	Total (%)
Overall Satisfaction from Child’s Perspective	Satisfied	60%
	Normal	40%
	Not satisfied	0%
Overall Satisfaction from Parent’s Perspective	Satisfied	73%
	Normal	26%
	Not satisfied	0%
Whether They Would Re-participate in NeuroWorld DTx Intervention	Yes	80%
	No	6.6%
	Other answer	13.3%

## Data Availability

Data are contained within the article.
